# Emotion regulation and delusion-proneness relate to empathetic tendencies in a transdiagnostic sample

**DOI:** 10.3389/fpsyt.2022.992757

**Published:** 2022-09-26

**Authors:** Emma N. Herms, Amanda R. Bolbecker, Krista M. Wisner

**Affiliations:** ^1^Department of Psychological and Brain Sciences, Indiana University, Bloomington, IN, United States; ^2^Program of Neuroscience, Indiana University, Bloomington, IN, United States

**Keywords:** empathy, perspective taking, emotion regulation, delusion-proneness, community sample

## Abstract

Empathetic tendencies (i.e., perspective taking and empathic concern) are a key factor in interpersonal relationships, which may be impacted by emotion regulation (i.e., reappraisal and suppression) and mental health symptoms, such as psychotic-like experiences. However, it is unclear if certain psychotic-like experiences, such as delusion-proneness, are still associated with reduced empathetic tendencies after accounting for emotion regulation style and dimensions of psychopathology that are often comorbid. In the current study, linear models tested these associations in a transdiagnostic community sample (*N* = 128), using the Interpersonal Reactivity Index (IRI), Emotion Regulation Questionnaire, and the Peter’s Delusion Inventory. Results indicated that perspective taking was positively associated with reappraisal and negatively associated with delusion-proneness, after controlling for age, sex, race, intelligence, and symptoms of anxiety and depression. A significant change in *R*^2^ supported the addition of delusion-proneness in this model. Specificity analyses demonstrated perspective taking was also negatively associated with suppression, but this relationship did not remain after accounting for the effects of reappraisal and delusion-proneness. Additional specificity analyses found no association between empathic concern and reappraisal or delusion-proneness but replicated previous findings that empathic concern was negatively associated with suppression. Taken together, delusion-proneness accounts for unique variance in perspective taking, which can inform future experimental research and may have important implications for psychosocial interventions.

## Introduction

Empathy is an integral part of interpersonal relationships (2), as it allows individuals to understand and respond to the affective experiences of others. Both emotion regulation style and mental health symptoms appear to impact one’s ability to engage in empathy. Nonetheless, the contribution of these multifaceted variables when considered simultaneously are crucial for understanding empathy variation informed by comorbidity perspectives of dimensional psychopathology in community samples. In the case of emotion regulation, the ability to reframe the situation and thereby regulate one’s own emotions during an interpersonal conflict allows space for empathy, such as understanding the other person’s point of view (3). Importantly, this may be difficult for persons experiencing mental health symptoms. Of particular interest to the current study is to examine the relationship between delusion-proneness, and empathetic tendencies. Critically, other mental health symptoms, such as anxiety and depression, are often comorbid with psychotic-like experiences in the general population (4–6) and have been separately associated with empathy e.g., (7, 8). It is therefore important to identify whether delusion-proneness is associated with empathetic tendencies after accounting for related phenomena, specifically emotion regulation and comorbid psychopathology. By clarifying this relationship, the current study aims to reveal unique contribution of delusion-proneness to reduced empathic tendencies in a transdiagnostic community sample and corroborate earlier psychosis-spectrum research on empathy.

Empathy is a multifaced construct (9), which goes beyond sharing an emotional state with another person. Within this construct it is important to delineate the cognitive and affective subdomains of empathy. *Cognitive empathy* represents the ability to infer the affective states of others, without necessarily sharing that emotion (10). While *Affective empathy* refers to sharing the real or perceived emotional state of others (10). A commonly used measure of empathy is the Interpersonal Reactivity Index (IRI). The subscales of the IRI that map onto cognitive and affective empathy are perspective taking and empathic concern, respectively (11, 12). Notably, emotion regulation is an important skill that can facilitate empathy (13), especially during interpersonal conflict (3). Emotion regulation includes several strategies to change what, when, or how an emotion is experienced. Cognitive *reappraisal* is a cognitive change strategy where an individual reframes an emotion-eliciting situation to alter the affective response (14). Expressive *suppression* is a response modulation strategy, where an individual inhibits any subsequent experience or behavior (e.g., facial expressions) during an affective response (14). Reappraisal and suppression have been shown to differentially impact empathetic tendencies in a variety of studies. Participants instructed to use reappraisal while reading about distressing events used more compassionate descriptors (as a measure of empathy) vs. when instructed to engage in suppression (15). Additionally, self-report measures of empathy (e.g., IRI), have illustrated positive relationships between reappraisal and cognitive empathy; whereas negative relationships between suppression and both cognitive and affective empathy have been observed (16, 17). However, there are some inconsistencies.

Laghi et al. (18) reported positive relationships between reappraisal and both perspective taking and empathic concern from the IRI, and they observed no relationships between suppression and any IRI subscales. Subsequent work also failed to identify differences in how individuals rated themselves on IRI subscales when stratified by high/low reappraisal use (19). Given these inconsistent findings, relationships between specific emotion regulation strategies and subdomains of empathy need further examination, especially while considering the role of mental health symptoms.

While mental health symptoms of anxiety and depression are more common in the general population (20), unique interpersonal difficulties have been observed for individuals living with psychosis e.g., (21, 22). Across the psychosis spectrum, there is ample evidence of deficits in cognitive and affective empathy (8, 23–25). However, it remains unclear if such deficits are specifically linked to psychotic-like symptoms, or if instead the deficits can be accounted for by symptoms of anxiety and depression which tend to co-occur with psychosis (4, 5, 26). Notably, previous studies in schizophrenia samples have demonstrated poorer self-reported perspective taking was associated with greater positive symptoms (27, 28); though findings have been inconsistent (29, 30). Of particular interest to the current study is subclinical variation in delusion, which can influence one’s beliefs about others and negatively impact social functioning (31). Around 10% of the general population report psychotic-like experiences during their lifetime (32, 33). Therefore, a community sample is ideal to extend previous work and clarify how dimensional variation in delusion-proneness relates to empathetic tendencies. The current study is specifically interested in the relationship between delusion-proneness and perspective taking, given findings of poorer cognitive but not affective empathy in schizophrenia samples (25). Critically, no previous study has examined the impact of delusion-proneness on empathetic tendencies while also controlling for demographic variables, intelligence, emotion regulation style, and comorbid mental health symptoms (i.e., anxiety and depression). This is an important next step to clarity effects given the inter-relatedness of multifaceted symptoms and cognitive factors existing across various clinical and non-clinical samples.

To address these knowledge gaps, the current study examines how emotion regulation strategies and delusion-proneness relate to empathetic tendencies, while controlling for demographic characteristics, intelligence, and comorbid mental health symptoms, in a transdiagnostic community sample. We hypothesize that decreased use of reappraisal and increased delusion-proneness will predict poorer perspective taking tendencies, which would highlight a unique role of psychotic-like experiences in cognitive empathy. To test specificity of these hypothesized associations, we assess parallel models employing empathic concern and suppression. The intention of the current study is to explore these processes using commonly employed surveys to inform future experimental research on examining mechanisms of interpersonal dysfunction in the psychosis spectrum.

## Materials and methods

### Participants

Data were utilized from participants between 18 and 60 years of age in the Nathan Kline Institute Rockland Sample (NKI-RS). This large, publicly available dataset was collected in Rockland County, New York, with details published elsewhere (1). To be included in the current study, participants needed to complete demographic surveys, intelligence testing, diagnostic assessments, and self-report questionnaires (described below). A total of 128 participants (98 female, mean age of 51.36) completed all measures of interest. Two participants had scores on a key measure greater than 3 standard deviations above the mean; however, results did not change upon exclusion. Therefore, results are reported using the full sample. A community sample was recruited for NKI-RS, meaning participants were not excluded based on physical or mental health. A Structured Clinical Interview of the DSM-IV-TR was conducted, and a total of 62 participants met or had previously met criteria for a mood or anxiety disorder (27), substance use disorder (11), post-traumatic stress disorder (1) or more than one of the previous diagnostic categories (23); but none met criteria for a psychotic or bipolar disorder. Thus 48% of this community sample met or had previously met criteria for a clinical disorder. By using the entire sample as a unified group, we capture a large range of psychopathology which reflects typical diagnostic rates of the general population in the United States (34). Notably, the self-report measures described below were completed by all individuals, and thus employed to capture the dimensional nature of their constructs (including dimensional psychopathology), which has benefits over categorical diagnostic labels for our investigation.

### Assessments

#### Interpersonal reactivity index

The IRI includes 28 self-report items, composing four subscales [7 items each; (35, 36)]. The current study focuses on two subscales: perspective taking (e.g., “I try to look at everybody’s side of a disagreement before I make a decision”) and empathic concern (e.g., “I often have tender, concerned feelings for people less fortunate than me”). The two additional subscales (fantasy and personal distress) have questionable validity (37, 38), and measure constructs beyond empathy (39, 40). Wang et al. (12) demonstrated strong performance of an IRI bifactor model, where perspective taking represents cognitive empathy and empathic concern represents affective empathy. The perspective taking and empathic concern subscales also have good reliability and validity e.g., (11, 41).

#### Emotion regulation questionnaire)

The Emotion regulation questionnaire [ERQ; (42)] includes 10 self-report items composing two subscales: reappraisal (6 items, including “When I’m faced with a stressful situation, I make myself think about it in a way that helps me stay calm”) and suppression (4 items, including “I control my emotions by not expressing them.”) The two-factor structure of the ERQ has been replicated in community samples and has good reliability and validity e.g., (43, 44).

#### Peter’s delusion inventory-21

The Peter’s delusion inventory-21 [PDI; (45)] is a measure of delusion-proneness that includes 21 self-report items assessing presence of delusional beliefs (e.g., “Do you ever feel as if things in magazines or on TV were written especially for you?”). If a belief is endorsed “yes,” participants then provide three separate ratings for distress, preoccupation, and conviction. The PDI was designed for the general population, and the total score (summation of number of endorsed delusional belief and associated distress, preoccupation, and conviction ratings) has been used to differentiate psychotic and non-psychotic populations [e.g., (46, 47)]. Good test-retest reliability, and concurrent validity have been reported [e.g., (45, 46)].

#### State Trait Anxiety Inventory

The State Trait Anxiety Inventory [STAI; (48)] includes 40 self-report items composing two subscales (20 items each) for state and trait anxiety. The STAI has adequate reliability [e.g., (49)].

#### Ruminative Responses Scale

The Ruminative Responses Scale [RRS; (50)] includes 22 self-report items that compose 3 subscales (Depression: 12 items, Brooding: 5 items, and Rumination: 5 items). The RRS has good reliability [e.g., (51)].

#### Wechsler abbreviated scale of intelligence

The Wechsler abbreviated scale of intelligence [WASI–II; (52)] is a general intelligence test composed of four subtests: Vocabulary, Block Design, Similarities, and Matrix Reasoning. The current study utilized the Full-Scale Intelligence Quotient (FSIQ-4) resulting from the combination of subscales.

### Analysis

Data analysis was performed in R (version 4.0.5). Self-report scales were assessed for normality using Shapiro-Wilk normality tests, and transformations were applied as needed (bestNormalize package). Skewness and Kurtosis were subsequently assessed (moments package) to identify any continued significant deviations (see Table 1). Statistical analyses include two-sided *t*-tests, Pearson correlations, Spearman correlations, and general linear models. Given this sample is predominately female, two sample *t*-tests (variance not assumed to be equal) were performed to assess for potential sex differences. General linear models were used to test the main hypothesis and for specificity analyses. Following the analyses, normality of the residuals (Q-Q plots) was examined to ensure adequacy and robustness of linear statistical methods.

**TABLE 1:** Sample characteristics and normality assessment of key variables.

	Mean (SD) or %	Range	Shaprio-Wilk normality test	Transformation	Skewness	Kurtosis
Age	51.36 (6.31)	39–60	*p* < 0.01	OQN	–0.06	2.54
Sex	77% female					
Race	84% white					
WASI-II	105.81 (13.26)	79–139	*p* = 0.56		–0.32*	1.82*
IRI-perspective taking	16.50 (4.07)	3–24	*p* < 0.01	SBC	–0.08	2.55
IRI-empathic concern	21.43 (4.50)	6–28	*p* < 0.01	Mean centered	–0.57	3.02
ERQ-reappraisal	29.34 (6.94)	8–42	*p* = 0.02	SBC	–0.02	2.95
ERQ-suppression	11.48 (4.78)	4–22	*p* < 0.01	OQN	0.11	2.52
PDI-total score	20.20 (26.41)	0–181	*p* < 0.01	SSR	0.54	3.08
STAI-TTAS	34.79 (10.89)	20–77	*p* < 0.01	AT	0.40	2.61
RRS-DS	18.23 (6.18)	12–48	*p* < 0.01	OQN	0.23	2.52

WASI–II, Wechsler Abbreviated Scale of Intelligence-II; IRI, Interpersonal Reactivity Index; ERQ, Emotion Regulation Questionnaire; PDI, Peter’s Delusion Inventory; STAI-TTS, State Trait Anxiety Inventory-Total Trait Anxiety Score; RRS-DS, Ruminative Response Scale-Depression Score; OQN, Ordered Quantile Normalization; SBC, Standardized Box Cox; SSR, Standardized Square-Root; AT, Arcsine Transformation. *The variable was not transformed, original skewness and kurtosis are reported.

## Results

### Descriptive statistics and correlations

Descriptive statistics for demographic variables and self-report measures are presented in Table 1. There were no significant differences across sex in age, intelligence, perspective taking, delusion-proneness, suppression, or anxiety and depression symptoms. However, in line with previous reports we observed elevated empathic concern and use of reappraisal in females (18, 53, 54). To facilitate interpretation of the linear models, Pearson correlations on transformed data were completed among the variables of interest (Table 2). Spearman correlations on non-transformed data were also computed and provided in Supplementary Table 1; importantly the relationships between the variables did not change.

**TABLE 2:** Correlations among model variables of interest.

	1	2	3	4	5
1.IRI-perspective taking	1				
2.IRI-empathic concern	0.554***	1			
3.ERQ-reappraisal	0.313***	0.181*	1		
4.ERQ-suppression	–0.224*	–0.332***	–0.252**	1	
5.PDI-total score	–0.181*	–0.018	0.141	–0.096	1

*Correlation is significant at 0.05 level. **Correlation is significant at 0.01 level. ***Correlation is significant at 0.001 level. IRI, Interpersonal Reactivity Index; ERQ, Emotion Regulation Questionnaire; PDI, Peter’s Delusion Inventory.

### Linear models

**FIGURE 1:**
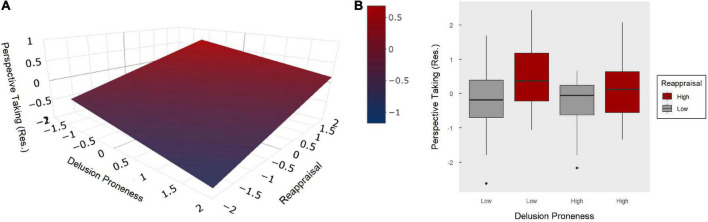
Model 1: Main effects of reappraisal and delusion-proneness on perspective taking. **(A)** Graph illustrating the effects of reappraisal and delusion-proneness (both z-scored) as a linear combination predicting perspective taking. Perspective taking was residualized to remove the variance explained by covariates of non-interest (i.e., age, sex, race, intelligence, anxiety, and depression). **(B)** Boxplot of the median split for delusion-proneness and reappraisal demonstrating no interaction between these two predictors on perspective taking (residualized).

All models include the following covariates of non-interest: age, sex, race, intelligence (WASI-II), as well as symptoms of anxiety (STAI-TTAS) and depression (RSS-DS). However, when demographic variables are not included in the models (i.e., sex, age, and WASI–II) model fit and relationships between variables were not meaningfully changed. To evaluate our key hypothesis, a linear model estimated the dependent variable of perspective taking as the linear combination of reappraisal, delusion-proneness, and covariates of non-interest (model 1; Table 3). Results revealed a significant overall model [*F* (119) = 5.17, *p* < 0.001], with significant main effects for reappraisal (β = 0.29, *p* = 0.001) and delusion-proneness (β = –0.23, *p* = 0.011; Figure 1A). A follow-up exploratory model showed a non-significant interaction between reappraisal and delusion-proneness (β < 0.01, *p* = 0.965), while the main effects remained significant and on par with model 1 (Figure 1B). Interestingly, the beta coefficient for the effect of delusion-proneness on perspective taking was larger in the linear model than it’s corresponding pairwise correlation with perspective taking (*r* = –0.18, *p* = 0.041, Table 2), suggesting a potential suppression effect (55). To examine this further, two sub-models were created with perspective taking estimated by reappraisal (model 1.A) or delusion-proneness (model 1.B) separately and coefficients were compared across the three models. Both sub-models were significant [*F* (120) = 4.73, *p* < 0.001; *F* (120) = 3.93, *p* < 0.001]; however, the coefficients for reappraisal (β = 0.24, *p* = 0.006) and delusion-proneness (β = –0.16, *p* = 0.079) were attenuated in these sub-models compared to the initial model that included both of these predictors (model 1). This confirmed a mild mutual suppression effect between reappraisal and delusion-proneness, wherein their beta coefficients are larger when both variables are included in the same model (55). To clarify, the observed mutual suppression is due to a positive relationship between the two predictor variables (although non-significant in this case), which have opposite relationships with the outcome variable (perspective taking). Having both reappraisal and delusion-proneness in the same model captures this underlying component that the two predictors share (e.g., shared variance), accentuating their respective inverse relationships with perspective taking. Importantly, a change in F-test between model 1 and model 1.A (where delusion-proneness was removed) yielded a significant change in *R*^2^, such that including delusion-proneness in the model led to a meaningful increase in variance explained in the prediction of perspective taking (*F* = 5.28, *p* = 0.011, delta *R*^2^ = 0.042).

**TABLE 3:** Subset of linear models testing effects of emotion regulation strategies and delusion-proneness on empathic tendencies.

	Mode 1: IRI-perspective taking				Model 4: IRI-empathic concern		
			
Predictor	β (*SE*)	*t*	*p*	Predictor	β (*SE*)	*t*	*p*
ERQ-reappraisal	0.29 (0.09)	3.38	0.001	ERQ-suppression	–0.31 (0.09)	–3.63	<0.001
PDI-total score	–0.23 (0.09)	–2.58	0.011	PDI-total score	–0.09 (0.09)	–0.97	0.333
STAI-TTAS	–0.47 (0.13)	–3.63	<0.001	STAI-TTAS	–0.36 (0.14)	–2.67	0.009
RRS-DS	0.45 (0.14)	3.07	0.003	RRS-DS	0.35 (0.14)	2.45	0.016
Age	–0.04 (0.08)	–0.55	0.582	Age	0.01 (0.09)	0.12	0.907
Sex	0.18 (0.20)	0.89	0.373	Sex	0.29 (0.20)	1.43	0.156
Race	–0.49 (0.23)	–2.16	0.033	Race	–0.13 (0.24)	–0.53	0.596
WASI-II	0.07 (0.08)	0.91	0.366	WASI-II	–0.07 (0.09)	–0.87	0.386
Overall model	Adj. *R*^2^ = 0.21	*F*_(8,119)_ = 5.17	<0.001	Overall model	Adj. *R*^2^ = 0.14	*F*_(8,119)_ = 3.52	0.001

IRI, Interpersonal Reactivity Index; ERQ, Emotion Regulation Questionnaire; PDI, Peter’s Delusion Inventory; STAI-TTS, State Trait Anxiety Inventory-Total Trait Anxiety Score; RRS-DS, Ruminative Response Scale-Depression Score; WASI–II, Wechsler Abbreviated Scale of Intelligence-II.

To evaluate the specificity of our main hypothesis, follow-up models were completed. In model 2 (see Supplementary Table 2), perspective taking was estimated as the linear combination of suppression and delusion-proneness. Results revealed a significant overall model [*F* (119) = 4.22, *p* < 0.001], with significant main effects for suppression (β = –0.19, *p* = 0.023) and delusion-proneness (β = –0.19, *p* = 0.036). To examine further, we added suppression to model 1, such that perspective taking was now estimated as the linear combination of reappraisal, suppression, and delusion-proneness. Results revealed a significant overall model [*F* (118) = 5.06, *p* < 0.001], with significant main effects for reappraisal (β = 0.27, *p* = 0.003) and delusion-proneness (β = –0.25, *p* = 0.006), but not suppression (β = –0.15, *p* = 0.068). Furthermore, a change in F-test indicated a non-significant increase in variance explained when adding suppression to model 1 (*F* = 2.63, *p* = 0.068, delta *R*^2^ = 0.021). Thus, confirming reappraisal and delusion-proneness seem to be the key predictors of perspective taking.

Additional specificity tests examined empathic concern in place of perspective taking. In model 3 (see Supplementary Table 2), empathic concern was estimated as the linear combination of reappraisal and delusion-proneness. The overall model was not significant [*F* (119) = 1.94, *p* = 0.060] and there were no significant main effects of reappraisal (β = 0.13, *p* = 0.183) or delusion-proneness (β = –0.07, *p* = 0.477). Finally, model 4 (Table 3) estimated empathic concern as the linear combination of suppression and delusion-proneness. Results revealed a significant overall model [*F* (119) = 3.52, *p* = 0.001] with a significant main effect of suppression (β = –0.31, *p* < 0.001), but not delusion-proneness (β = –0.090, *p* = 0.333).

## Discussion

This is the first study to our knowledge to examine relationships between delusion-proneness and empathetic tendencies while controlling for inter-related factors, including emotion regulation and comorbid mental health symptoms. Our findings suggest individuals who report decreased use of reappraisal and increased delusion-proneness are more likely to have impaired perspective taking tendencies. This community-based study adds to the existing literature by revealing the negative impact of delusion-proneness on perspective taking above and beyond the effects of reappraisal, intelligence, demographic variables, and internalizing psychopathology. That is, delusion-proneness remained clinically and statistically relevant when including other important predictors in the model. Specifically, a medium effect size remained for delusion-proneness alongside a large effect size for internalizing psychopathology and a medium effect size for reappraisal, when included in the model simultaneously.

Findings from the current study aligns with previous work utilizing the IRI in psychotic samples, wherein lower perspective taking but not empathic concern was observed in individuals with schizophrenia (25), and negative correlations were observed between perspective taking and positive symptoms in schizophrenia (27, 28). Our findings contradict one previous positive correlation between perspective taking and the magical ideation subscale of the Schizotypal Personality Questionnaire (SPQ) in an undergraduate sample (39). However, that study had inconsistent findings, with no correlations between SPQ subscales of suspiciousness or ideas of reference and IRI subscales. While replication of the current study is needed, our transdiagnostic community sample may be better equipped to identify robust associations between delusion-proneness and IRI subscales due to a broader representation of dimensional psychopathology. In summary, our findings not only converge with major trends in the literature but also highlight the unique negative impact of delusion-proneness on perspective taking, which has implications for interpersonal relationships and highlights potential opportunities for future psychosocial interventions.

In specificity analyses perspective taking initially appeared to have a negative association with suppression. However, this relationship did not remain after accounting for the effects of reappraisal and delusion-proneness in the same model, further solidifying the key finding described above. In contrast, suppression appeared to be a unique negative predictor of empathic concern, as neither reappraisal nor delusion-proneness were associated with this IRI subscale. This replicates previous findings between suppression and empathetic tendencies (16, 17). Specifically, a medium effect size was observed for suppression, along with large effect sizes for internalizing psychopathology. We speculate the negative association reflects the nature of suppression. If an individual typically limits expression of their own affective experience (suppression), they might (consciously or subconsciously) inhibit their affective responses to another’s emotional state, which is needed for empathic concern. Overall, findings align with previous studies where reappraisal and suppression have opposite effects on empathetic tendencies (15–17).

### Limitations and future directions

Self-report measures are a limitation of the current study, as they are dependent on self-awareness, and limit the examination of mechanisms. However, task-based literature can be combined with the current findings to inform future experimental research that may extend this line of work. One task appearing relevant is “Reading the Mind in the Eyes” (Eyes Test), a classic Theory of Mind paradigm (ToM; ability to differentiate between one’s own and other’s mental state) with relationships observed across psychopathology. While ToM has been used interchangeably with cognitive empathy (10, 56, 57), studies assessing the relationship between performance on the Eyes Test and subscales of the IRI have found no associations (27, 58, 59). Thus, researchers concluded the two constructs are related but separate (39, 59). An experimental paradigm that might more specifically target the relationship between delusion-proneness and perspective taking is the Minnesota Trust Game (MTG). The MTG is a social-economic decision-making paradigm designed to distinguish suspiciousness driven mistrust (when a partner has no incentive to betray you) and rational mistrust (when a partner is incentivized to betray you), which requires attributing partner intentions (60). Dimensional variation in persecutory ideation has been specifically linked to behavioral indices of suspiciousness using the MTG (60, 61). Thus, in future studies we plan to administer the IRI alongside the MTG, to further investigate associations between delusion-proneness and perspective taking using a multitrait-multimethod approach.

Future studies would also benefit from including a measure that captures the quantity and quality of interpersonal relationships or socialization more broadly, which was not available in the NKI dataset. Such a measure could help to further characterize interpersonal functioning and thereby compliment the empathy assessments from the IRI, as well as provide capability to examine whether empathic tendencies from the IRI might mediate the relationship between psychotic-like experiences and broader measures of interpersonal functioning in community samples. Additional limitations of the current study include the lack of a negative symptoms (e.g., avolition or flat affect) measure in the NKI dataset to assess associations with perspective taking or empathic concern. Previous work has identified relationships between the negative symptom approximation from the SPQ and empathy [primarily driven by empathic concern on the IRI; (39)] as well as reduced affective empathy specifically (62, 63). Lastly, the sample composition in the current study included a large proportion of middle-aged female Caucasian participants; future work is needed to replicate our findings in a more gender balanced and diverse sample.

## Conclusion

This community-based study clarifies the literature by revealing that individuals who report increased delusion-proneness are more likely to have impaired perspective taking abilities during interpersonal interactions, even after accounting for reappraisal style, and individual differences in intelligence, demographic variables, and internalizing psychopathology. Furthermore, the negative impact of delusion-proneness appears specific to cognitive empathy (i.e., perspective taking). The current study informs future experimental work on mechanisms underlying interpersonal dysfunction and may have important implications for psychosocial interventions across clinical and non-clinical samples.

## Data availability statement

Publicly available datasets were analyzed in this study. This data can be found here: https://data.rocklandsample.rfmh.org.

## Ethics statement

The studies involving human participants were reviewed and approved by Indiana University Bloomington (IRB #2009962152) for secondary data analysis. The initial Institutional Review Board approval was obtained by the Nathan Kline Institute see Nooner et al. (1). The patients/participants provided their written informed consent to participate in this study.

## Author contributions

EH: conceptualization, formal analysis, visualization, writing—original draft, and writing—review and editing. AB: writing—review and editing. KW: conceptualization, project administration, supervision, and writing—review and editing. All authors contributed to the article and approved the submitted version.
