# Supplementary material for: Emotion regulation and delusion-proneness relate to empathetic tendencies in a transdiagnostic sample
Source: Front Psychiatry. 2022 Sep 26;13:992757. doi: 10.3389/fpsyt.2022.992757 (PMC9548608; doi:10.3389/fpsyt.2022.992757)
Supplement: Supplementary file 1 [file Data_Sheet_1.docx]

| Supplementary Table 1  Spearman correlations using non-transformed variables of interest | | | | | |
| --- | --- | --- | --- | --- | --- |
|  | 1 | 2 | 3 | 4 | 5 |
| 1.IRI-Perspective Taking | 1 |  |  |  |  |
| 2.IRI-Empathic Concern | 0.551 ^***^ | 1 |  |  |  |
| 3.ERQ-reappraisal | 0.333 ^***^ | 0.201 ^*^ | 1 |  |  |
| 4.ERQ-suppression | -0.235 ^**^ | -0.337 ^***^ | -0.260^**^ | 1 |  |
| 5.PDI-total score | -0.174 ^*^ | -0.041 | .130 | -0.085 | 1 |
| * Correlation is significant at 0.05 level  ** Correlation is significant at 0.01 level  *** Correlation is significant at 0.001 level  *Note.* IRI = Interpersonal Reactivity Index, ERQ = Emotion Regulation Questionnaire, PDI = Peter’s Delusion Inventory | | | | | |

| Supplementary Table 2  Subset of linear models testing effects of emotion regulation strategies and delusion-proneness on empathic tendencies | | | | | | | | |  |
| --- | --- | --- | --- | --- | --- | --- | --- | --- | --- |
|  | | Model 2: IRI-Perspective Taking | | |  | Mode 3: IRI-Empathic Concern | | |  |
| Predictor | β (*SE*) | | *t* | *p* | Predictor | β (*SE*) | *t* | *p* | |
| ERQ-Suppression | -0.19 (0.08) | | -2.30 | 0.023 | ERQ-Reappraisal | 0.13 (0.09) | 1.34 | 0.183 | |
| PDI-Total Score | -0.19 (0.09) | | -2.13 | 0.036 | PDI-Total Score | -0.07 (0.10) | -0.71 | 0.477 | |
| STAI-TTAS | -0.52 (0.13) | | -3.93 | <0.001 | STAI-TTAS | -0.37 (0.14) | -2.59 | 0.011 | |
| RRS-DS | 0.41 (0.14) | | 2.92 | 0.004 | RRS-DS | 0.35 (0.15) | 2.38 | 0.019 | |
| Age | -0.05 (0.08) | | -0.57 | 0.571 | Age | -0.03 (0.09) | -0.34 | 0.731 | |
| Sex | 0.29 (0.20) | | 1.45 | 0.149 | Sex | 0.32 (0.22) | 1.50 | 0.136 | |
| Race | -0.43 (0.23) | | -1.86 | 0.066 | Race | -0.13 (0.25) | -0.52 | 0.603 | |
| WASI-II | 0.08 (0.08) | | 0.99 | 0.323 | WASI-II | -0.05 (0.09) | -0.60 | 0.548 | |
| Overall Model | Adj. *R*^2^ = 0.17 | | *F*_(8,119)_ = 4.22 | <0.001 | Overall Model | Adj. *R*^2^ = 0.06 | *F*_(8,119)_ = 1.94 | 0.060 | |
| *Note.* IRI=Interpersonal Reactivity Index, ERQ=Emotion Regulation Questionnaire, PDI=Peter’s Delusion Inventory, STAI-TTS=State Trait Anxiety Inventory-Total Trait Anxiety Score, RRS-DS=Ruminative Response Scale-Depression Score, WASI=Wechsler Abbreviated Scale of Intelligence-II | | | | | | | | |  |
